# An Occlusive Form of Acute Gangrenous Appendicitis With Periappendicular Abscess in an Elderly Patient: A Case Report and Literature Review

**DOI:** 10.7759/cureus.36213

**Published:** 2023-03-16

**Authors:** Alfred Najm, Irina M Bejenaru, Stefania L Manolescu, Ramona Iliescu, Sanda Maria Cretoiu, Bogdan S Gaspar

**Affiliations:** 1 Surgery, Emergency Clinical Hospital/Carol Davila University of Medicine and Pharmacy, Bucharest, ROU; 2 Surgery Clinic, Emergency Clinical Hospital, Bucharest, ROU; 3 Surgery Clinic, Buzău Emergency County Hospital, Buzau, ROU; 4 Morphological Sciences, Cell and Molecular Biology and Histology, Carol Davila University of Medicine and Pharmacy, Bucharest, ROU

**Keywords:** appendicectomy, elderly patient, intestinal occlusion, exploratory laparotomy, computerized tomography

## Abstract

Acute appendicitis represents one of the common causes of admission to the emergency department. In rare cases, patients with appendicitis can suffer complications such as intestinal obstruction. These particular cases of occlusive appendicitis with a periappendicular abscess usually occur in elderly patients and can develop in an aggressive form, nonetheless with a favorable evolution. We present a case of an 80-year-old male patient, reporting symptoms similar to an occlusive digestive pathology: abdominal pain, intestinal transit disorders, and fecal vomiting. A computerized tomography scan suggested a mechanical bowel obstruction. The patient had an exploratory laparotomy indication to find the cause of the obstruction. The peritoneal cavity inspection revealed an occlusive form of acute gangrenous appendicitis with a periappendicular abscess. An appendectomy was performed. In conclusion, as surgeons, we must always take into consideration that acute appendicitis can represent a cause of intestinal obstruction, especially in elderly patients.

## Introduction

Acute appendicitis is considered one of the most common causes of acute abdominal pain, has a maximum incidence in men in the second or third decade of life, and is less often in the extremes of age [1]. Although the etiology of appendicitis is unclear, it seems that obstruction of the appendicular lumen is the most incriminated physiopathological mechanism [2]. Modern theories suggest some additional etiological factors such as allergies and microbiota changes [3].

The clinical features of acute appendicitis are periumbilical abdominal pain that migrates to the right iliac fossa in McBurney's point, anorexia, nausea, fever, and right iliac fossa tenderness [4]. Association between these symptoms and neutrophilic leucocytosis and ultrasound examination showing an inflamed appendix is sufficient to make a clinically positive diagnosis using the Alvarado score [5]. The gold standard treatment for simple acute appendicitis is appendicectomy associated with large-spectrum antibiotherapy [6]. Conservatory management is indicated for specific cases like inflammatory appendiceal mass and appendiceal abscess, and it consists of empiric antibiotic therapy, percutaneous drainage for abscesses that measure more than 4 cm, monitoring of vital signs and abdominal exam, colonoscopy after the acute episode in patient s > 40 years old in search of colonic malignancy [7].

The particularity of the case presented herein exists in the fact that, often, an intestinal occlusion can mask the clinical signs of acute appendicitis. Thus, in the case of elderly patients, preoperative diagnosis difficulties are frequently encountered requiring exploratory laparotomy. This particular case is interesting due to the discordance between the presumptive preoperative diagnosis, which would have been more suggestive of an intestinal occlusion caused by a tumor, rather than an acute gangrenous appendicitis with a periappendicular abscess that was found intraoperatively. Appendicectomy was performed in our Surgery Clinic, associating antibiotics for the abdominal contamination, with good postoperative evolution of the patient.

## Case presentation

An 80-year-old Caucasian patient was admitted to the emergency department after five days of continuous diffuse abdominal pain aggravated in the last 24 hours, progressive lack of intestinal transit for feces and gases, marked asthenia, and the appearance of fecal vomiting. The patient has a medical history of allergic asthma and non-steroidal anti-inflammatory drug (NSAID) intolerance, a surgical history of bilateral inguinal hernia operated on five years ago, with non-important family medical issues. The presumptive diagnosis at the admission was mechanical bowel obstruction suspecting a tumoral cause because of the advanced age. The patient mentioned that the symptoms started suddenly five days ago with an acute debut, were continuous and progressive during the next days, accompanied by nausea, loss of appetite, and absolute constipation. Symptomatology progressively accentuated and become aggravated by fecal vomiting.

Clinical examination at admission in the emergency department showed a conscious, hard-cooperating, temporally spatially oriented patient. Checking the vital signs we found his temperature was 36.7°C, pulse rate 98 beats/minute, blood pressure 100/60 mm Hg, respiratory rate 17 per minute, and BMI 24. The patient was pale, dehydrated, and with a generalized abdominal muscle contracture.

Physical examination showed abdominal distension, abdominal tenderness, positive Blumberg sign, and tenderness on percussion (positive Mandel sign) with an absence of bowel sound. A digital rectal examination revealed an empty rectum.

The laboratory tests of the patient showed mild leukocytosis 10,100 leukocytes/mm3 (neutrophils 1,92 x103/µl), glucose 132 mg/dl, and normal biochemistry. Platelet count was 275x103/µL, bleeding time 1 min, clotting time 3 min, active partial thromboplastin time (APTT) 32 sec, and fibrinogen 285 mg/dL. A plain abdominal X-ray describes important aerocolia and multiple enteral "air-fluid levels" with a variable location in the intestine. The pulmonary X-ray at admission was normal.

Summarizing the following elements of the medical case report - personal history, local abdominal examination, and paraclinical explorations (laboratory tests, abdominal and pulmonary X-ray examination) - we concluded an admission diagnosis of bowel obstruction.

Initial treatment was started in the surgery department that included: the insertion of a nasogastric tube (NT) followed by evacuation of approximately 300 ml of fecal liquid, fluid and electrolytic rebalancing, and antibiotic intravenous infusion with third-generation cephalosporins (cefoperazone and sulbactam) after testing the patient for antibiotic sensibility. An evacuation enema was performed as well but without effect.

**Figure 1:**
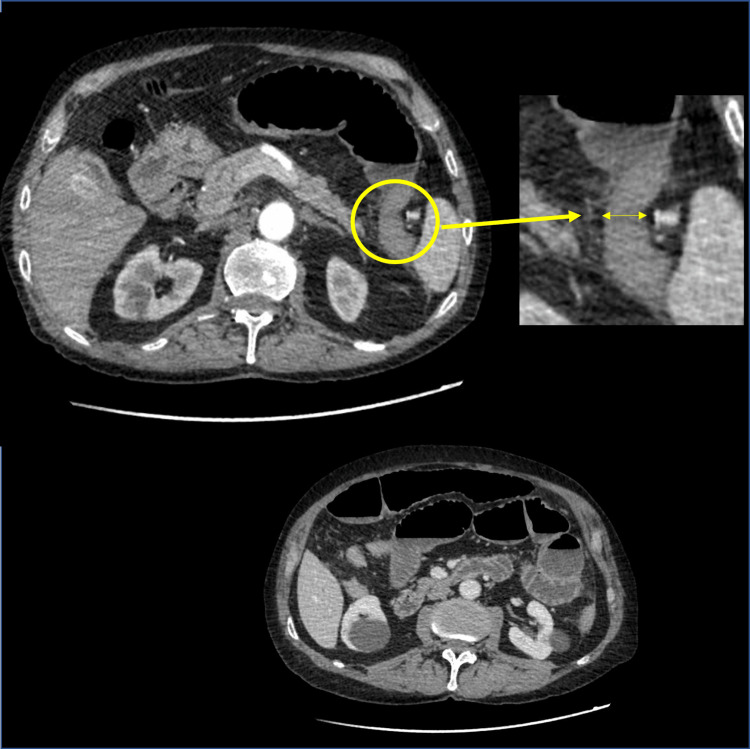
CT scan with a contrast-axial view A. Aspect of small bowel dilatation and depicting transverse colon-descending junction in relation to a dilated enteral loop (insert detail), without demarcation interface, in the left hypochondrium. B. Dilated bowel loops in the abdominal with horizontal hydro-aerial levels.

Surprisingly, the computerized tomography (CT) scan missed acute gangrenous appendicitis with a perpendicular abscess reported as an intraoperative finding. The abdominal and pelvic CT scan with contrast substance injection showed dilated bowel loops in the abdominal and pelvic cavity up to a maximum caliber of 5 cm, with mixed hydro-aerial content and present horizontal hydro-aerial levels: the ascending, transverse, and the descending colon with collapsed lumen, transverse colon descending junction in relation with a dilated enteral loop in the left hypochondrium, without a demarcation interface between them, with suspicion of adherence syndrome or volvulus (Figure 1). Presence of liquid in the pouch of Douglas with a maximum thickness of 2 cm thickness. The conclusion of the imaging exam was a mechanical bowel obstruction.

The preoperative care included: the correction of hydro-electrolytic imbalances and aspiration of gastric content. A blood sample was taken to determine the blood type and Rh and two units of blood were prepared. An indwelling urinary catheter was installed for urine output monitoring. An urgent anesthesia consultation was done establishing an American Society of Anesthesiologists (ASA) anesthetic risk scale of Class IV, which means severe, disabling, life-threatening systemic disease with a risk of 7.8% mortality requiring a gentle induction and good relaxation. Moreover, psychological care was needed in order to capture the confidence of the patient in the surgeon and in the surgical act and indication, as well for obtaining his consent for the surgical technique, after being properly informed about the possible postoperative risks. The laparoscopic approach was contraindicated by the anesthesiologist due to the age of the patient and his history of bronchial asthma.

**Figure 2:**
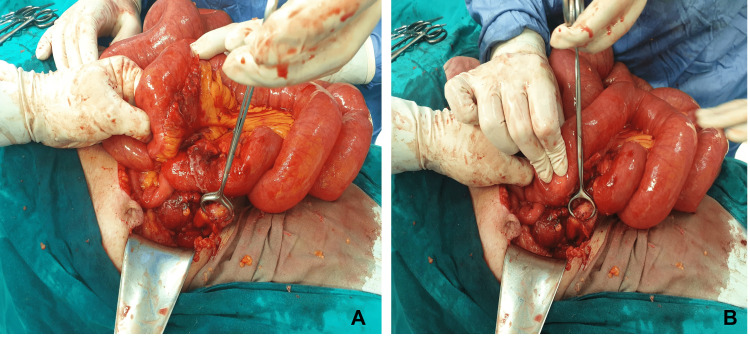
Intraoperative image of the gangrenous appendix and small bowel dilatation

The surgical approach was a median supra- and sub-umbilical incision prolonged caudally during the abdominal exploration. The peritoneal cavity inspection reveals a large volume of distended bowel loops; the exploration continued and an ileal loop is found attached in the right iliac fossa through its meso and a retrocecal abscess is detected. When exploring the peritoneal cavity, the rest of the viscera have a normal macroscopic appearance. After a laborious dissection of the affected region, one entered the abscess cavity where a gangrenous appendix is identified (Figure 2). Purulent fluid was collected for a bacteriological examination.

Appendectomy was performed by invaginating the appendicular stump with a monofilament suture of PDS 4/0. The following steps included: retrograde emptying of the intestinal loops, hemostasis control, and lavage of the interested area to limit bacterial contamination, followed by pelvic drainage. The abdominal wall was sutured with PDO 2. Skin sutures and wound dressing bandages were performed. The appendix with gangrenous macroscopic aspect was sent for the histopathological examination.

The postoperative positive diagnosis was an occlusive form of acute gangrenous appendicitis with a periappendicular abscess. The postoperative recovery was uneventful, and the patient was discharged home one week postoperatively.

The particularity of the case was the occlusive form of acute gangrenous appendicitis in an elderly patient with clear symptoms of high intestinal occlusion.

## Discussion

In elderly patients, it is more frequent to have complicated appendicitis like a periappendicular abscess, appendicular adenocarcinoma, inflammatory mass, or perforated appendicitis associated with occlusive form and bowel obstruction [8]. Perforated appendicitis increases mortality and morbidity in elderly subjects, increasing the risk of generalized peritonitis, sepsis, adhesion formation, and sometimes small bowel obstruction, which sometimes can be aggravated by personally associated comorbidities leading to severe decompensation [9,10]. A recent systematic review of the literature describes the incidence, form, and risk factors for perforation, mortality, and morbidity in the intestinal obstructive form of acute appendicitis (for details, see [9]).

Mechanical obstructions of the bowel can involve either the small intestine or the colon. The causes can be multiple, however, one can categorize them into intraluminal, intramural, or extramural origin [11,12]. Small bowel obstructions are frequently produced by intra-abdominal adhesions or hernias, whereas large bowel obstructions are more likely to be produced by tumors, volvulus, and strictures [13]. However, acute appendicitis has not been recognized as an etiologic factor in the vast majority of small bowel obstructions. Acute appendicitis, in either a simple or complicated form, is a ubiquitous problem, accounting for approximately 5% of all emergency department admissions for patients under 65 years and 30% of acute surgical abdominal emergencies in patients under 50 years old worldwide [14]. Small bowel obstruction due to acute appendicitis can be caused either by mechanical obstruction resulting from a peri-appendicular inflammation or can be the result of ileus due to localized peritonitis caused by the vermiform appendix perforation [15]. Sometimes, both instances can co-exist, however, the pathognomonic signs of acute appendicitis are masked by signs of intestinal obstruction, especially in an elderly patient [16].

This paper presents a particular case of an elderly patient with an occlusive form of perforated appendicitis who was hospitalized in the General Surgery Clinic of the Emergency Clinical Hospital of Bucharest. The peculiarity of the case lies in the fact that, due to the age criteria, we faced the problem of establishing a diagnosis of certainty, oscillating between an oncological cause of obstruction and the occlusive form of acute gangrenous appendicitis.

Clinical features of acute appendicitis are typically beginning with prodromal symptoms of anorexia, nausea, and progressive periumbilical pain migrating in the right iliac fossa. In uncomplicated forms, a low fever is associated, with mild leukocytosis. Complicated tforms, such as periappendicular abscess, perforated appendicitis with bowel obstruction due to general peritonitis, develop severe clinical features with increasing abdominal pain, peritoneal irritation, positive Blumberg sign, high fever, vomiting, digestive troubles, and even sepsis, which is a negative factor and increases morbidity and mortality, especially in elderly patients like this case [17].

Regarding the imaging exams, in the emergency department, abdominal ultrasound imaging has become the first investigation that can describe rapidly an appendicitis aspect, with fluid surrounding the appendix, lack of peristalsis, presence of an appendicolith, abscess, or even perforation showing free air in the right iliac fossa [18]. When there is a doubt or an emergency due to a septic condition, a CT scan can be performed to have higher specificity and sensitivity [19]. In our case, ultrasound was considered to bring less information than CT scan with contrast, taking into account the severe condition of the patient at admission. The imagistic exams like abdominal X-rays described important aerocolia and multiple enteral air-fluid levels. The CT scan with contrast was more specific showing an aspect of mechanical bowel obstruction.

For the presented case, a differential diagnosis was made after the CT scan exam, and other possibilities were mentioned: acute mesenteric ischemia, peritonitis by digestive perforation, adherence syndrome, diverticulitis, kidney colic, or acute pancreatitis. The first suspicion was intestinal obstruction and digestive perforation, so therapy with broad-spectrum antibiotics was instituted, and we took the decision to indicate a surgical exploration. Without the specific treatment, there could be consequences like the aggravation of hydro-electrolytic imbalances, sepsis, and lethal outcome.

Surgical treatment was decided for the present case in order to find the cause of the bowel obstruction by doing a median laparotomy. The intraoperative positive diagnosis was acute gangrenous perforated appendicitis, an occlusive form with very dilated bowel loops. Appendicectomy was performed, followed by abdominal cavity lavage, and pelvic drainage. The patient had a favorable evolution, and he was discharged one week after the surgery.

We examined the published literature and found that most of the published papers having an intestinal obstruction due to appendicitis as the subject were reports of clinical cases. These were analyzed in a systematic review by Makama et al. in 2017, who showed that appendicitis is an important cause of intestinal obstruction, but pre-operative diagnosis is still a major challenge in these cases [9]. We consider that the current presented case is unique since there is no similar case to present acute gangrenous appendicitis with a periappendicular abscess in an elderly patient. The most recently presented cases (only two in number) discuss intestinal obstruction due to appendiceal tourniquet or appendiceal knotting [15,20].

## Conclusions

In conclusion, even if appendicitis is more often encountered in young patients, we have to keep this diagnosis in mind for all ages because sometimes complicated appendicitis can be confused with a tumoral cause of bowel obstruction, and surgical treatment is imperative. Numerous cases of appendicitis, diagnosis algorithms, clinical score diagnosis, and risk factors of perforation are presented in the specialized literature, but the presented case has the particularity of an occlusive form of acute gangrenous appendicitis with a periappendicular abscess in an elderly patient. This case reminds us that a complicated form of appendicitis should be included in the differential diagnostic procedure related to occlusive episodes in elderly patients.
